# A phase 2 clinical trial of luspatercept in non-transfusion-dependent patients with myelodysplastic syndromes

**DOI:** 10.1007/s12185-024-03872-3

**Published:** 2024-11-21

**Authors:** Hiroshi Kosugi, Tomoaki Fujisaki, Hiromi Iwasaki, Atsushi Shinagawa, Hiroatsu Iida, Tatsuro Jo, Shiro Kubonishi, Yasuyoshi Morita, Yasuhiro Nakashima, Koichi Onodera, Kenshi Suzuki, Takahiro Suzuki, Yotaro Tamai, Kensuke Usuki, Akira Yokota, Hideyuki Yonaga, Jin Hayakawa, Shuichi Midorikawa, Mitsufumi Nishio, Makoto Suda, Kosei Matsue

**Affiliations:** 1 Department of Hematology, Ogaki Municipal Hospital, 4-86, Minaminokawacho, Ogaki 503-8502 Japan; 2 Department of Hematology, Matsuyama Red Cross Hospital, Matsuyama, Japan; 3 Department of Hematology, Clinical Research Institute, National Hospital Organization Kyushu Medical Center, Fukuoka, Japan; 4 Department of Internal Medicine, Hitachi General Hospital, Hitachi, Japan; 5 Department of Hematology, National Hospital Organization Nagoya Medical Center, Nagoya, Japan; 6 Department of Hematology, Japanese Red Cross Nagasaki Genbaku Hospital, Nagasaki, Japan; 7 Department of Hematology and Oncology, Japanese Red Cross Society Himeji Hospital, Himeji, Japan; 8 Department of Hematology and Rheumatology, Kindai University Hospital, Osakasayama, Japan; 9 Department of Hematology and Hematopoietic Cell Transplantation, Osaka Metropolitan University Hospital, Osaka, Japan; 10 Department of Hematology, Tohoku University Hospital, Sendai, Japan; 11 Department of Hematology, Japanese Red Cross Medical Center, Tokyo, Japan; 12 Department of Hematology, Kitasato University Hospital, Sagamihara, Japan; 13 Division of Hematology, Shonan Kamakura General Hospital, Kamakura, Japan; 14 Department of Hematology, NTT Medical Center Tokyo, Tokyo, Japan; 15 Department of Hematology, Chiba Aoba Municipal Hospital, Chiba, Japan; 16 Bristol Myers Squibb, Tokyo, Japan; 17 Division of Hematology/Oncology, Department of Internal Medicine, Kameda Medical Center, Kamogawa, Japan

**Keywords:** Anemia, Low risk, Luspatercept, Myelodysplastic syndromes, Non-transfusion dependent

## Abstract

Luspatercept has shown durable clinical efficacy for the treatment of anemia in transfusion-dependent patients with lower-risk myelodysplastic syndromes (LR-MDS). We report the results of a prespecified primary analysis of a phase 2 trial of luspatercept in non-transfusion-dependent (NTD) Japanese patients with anemia due to LR-MDS. Luspatercept (starting dose 1.0 mg/kg) was administered subcutaneously once every 3 weeks. The primary endpoint was the proportion of patients who achieved hematological improvement-erythroid (HI-E) response (≥ 1.5 g/dL increase in hemoglobin level for 8 weeks) without transfusions within the first 24 weeks of treatment. At the primary analysis data cutoff, 21 patients had been enrolled/treated; 17 and 10 patients had completed 24 and 48 weeks of treatment, respectively. HI-E response occurred within 24 weeks in 10 patients (47.6%; 95% confidence interval, 25.7–70.2; *P* < 0.0001), which was significantly higher than the predefined threshold (10%). By week 48, HI-E response occurred in 12 patients (57.1%) and 17 patients (81.0%) remained NTD. Luspatercept was well tolerated. Three patients (14.3%) had grade 3–4 treatment-related treatment-emergent adverse events. Luspatercept resulted in statistically and clinically significant improvements in hemoglobin levels, and may help delay the need for transfusions in NTD patients with LR-MDS.

**Supplementary Information:**

The online version contains supplementary material available at 10.1007/s12185-024-03872-3.

## Introduction

Myelodysplastic syndromes (MDS) are a diverse group of clonal hematopoietic stem cell neoplasms that predominantly affect the elderly, and are characterized by ineffective hematopoiesis, blood cytopenias (anemia, neutropenia, and/or thrombocytopenia) of varying severity, and potential to progression to acute myeloid leukemia (AML) [1–3]. Patients with lower-risk (LR)-MDS have a reduced likelihood of AML transformation, and typically have a long survival [4–6]. However, LR-MDS commonly manifests clinically as anemia, which can lead to falls, cognitive decline, cardiovascular morbidity, and a reduced quality of life [1, 4, 7]. Effective treatment of anemia is therefore essential for the maintenance of overall health and quality of life [4, 8]. For patients with LR-MDS, the main goals of treatment are to ameliorate anemia and prevent red blood cell (RBC) transfusion dependence, a prognostic factor with a negative impact on quality of life [4, 5, 8].

In non-transfusion-dependent (NTD) patients with LR-MDS, early initiation of active treatment for symptomatic anemia can prevent or delay the progression to RBC transfusion dependence, and potentially improve overall survival [9, 10]. Erythropoiesis-stimulating agents (ESAs), such as darbepoetin-α and epoetin-α, are the guideline-recommended, first-line standard-of-care for the treatment of symptomatic anemia in patients with LR-MDS and serum erythropoietin (EPO) ≤ 500 U/L [8, 11, 12]. In clinical studies, including randomized controlled trials, ESAs consistently improved erythroid response rates in transfusion-dependent (TD) and NTD patients with LR-MDS [13–15], but primary resistance and loss of response to ESAs are common [6]. Patients with serum EPO levels of 200–500 U/L are less likely to respond to ESAs than patients with EPO levels of < 200 U/L and are therefore more likely to require alternative treatments [16]. However, other treatment options for NTD patients with LR-MDS are limited, with most patients eventually requiring long-term RBC transfusions [6, 8].

Luspatercept, an erythroid maturation agent, is a recombinant fusion protein that acts as a ligand trap for select transforming growth factor-β superfamily ligands to decrease SMAD2/3 signaling, thereby promoting erythroid maturation via late-stage erythroblast expansion and differentiation [17–19]. In mouse models of MDS, treatment with RAP-536 (the mouse analog of luspatercept) reduced erythroid hyperplasia, enhanced erythropoiesis, and increased RBC counts and hemoglobin levels [19].

The efficacy and safety of luspatercept in TD patients with LR-MDS has been assessed in two phase 3 clinical trials (MEDALIST and COMMANDS) [20, 21]. The double-blind, placebo-controlled MEDALIST trial demonstrated that, compared with placebo, Iuspatercept reduced the severity of anemia and increased the proportion of patients achieving transfusion independence in TD patients with LR-MDS with ring sideroblasts (RS) who were refractory to or unlikely to respond to ESAs, or who had discontinued ESA treatment previously because of an adverse event [20]. In the open-label, randomized COMMANDS trial greater efficacy was seen with the use of luspatercept than with epoetin-α in ESA-naïve TD patients with LR-MDS [21]. Furthermore, the safety profile of luspatercept in the COMMANDS trial was generally consistent with its known safety profile and with previous trials, including the MEDALIST trial.

A subgroup analysis of long-term results of the phase 2 PACE-MDS trial, which enrolled patients with LR-MDS regardless of transfusion burden, has indicated that luspatercept may be particularly effective in NTD patients [22]. Here, we report the results of a prespecified primary analysis of an phase 2 trial evaluating the efficacy, safety, and pharmacokinetics of luspatercept specifically in NTD Japanese patients with anemia caused by LR-MDS.

## Materials and methods

### Study design and oversight

This phase 2, multicenter, single-arm trial was conducted at 16 clinical sites in Japan. As shown in supplemental Fig. 1, the trial comprised a screening period, treatment period, and post-treatment follow-up period, including 42-day safety and long-term follow-up.

**Fig. 1:**
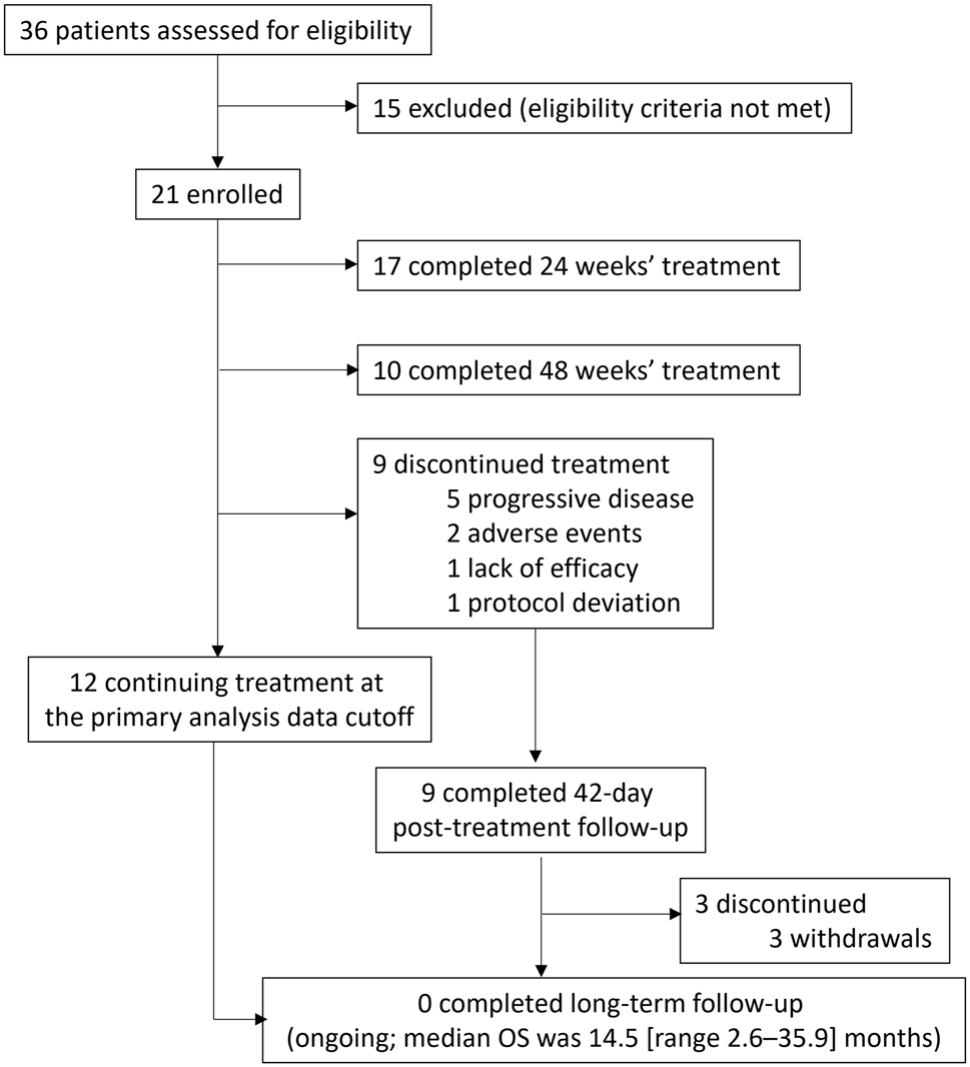
Patient disposition. OS, overall survival

The study was conducted in accordance with the Good Clinical Practice Guideline E6 from the International Council for Harmonization of Technical Requirements for Pharmaceuticals for Human Use, the general ethical principles outlined in the Declaration of Helsinki, and applicable national, prefectural, and local laws. All patients provided written informed consent. The study protocol was approved by the institutional review board or independent ethics committee at each clinical site. The study was registered at www.ClinicalTrials.gov on 3 April 2019 (NCT03900715).

### Patients

Eligible patients were aged ≥ 20 years and had MDS diagnosed according to the World Health Organization (WHO) 2016 criteria [23], with < 5% bone marrow blasts. They had LR-MDS, defined according to the revised International Prognostic Scoring System (IPSS-R) as disease of very low, low, or intermediate risk [24]. Patients had symptomatic anemia with a mean hemoglobin level < 10.0 g/dL (from two measurements, one performed 7–35 days before the first dose of luspatercept and one within 1 day prior to the first dose of luspatercept), and no requirement for RBC transfusions (no transfusions ≤ 16 weeks before the first dose of luspatercept). Previous ESA use, administered ≥ 8 weeks before the first dose of luspatercept, was permitted. Patients were required to have an Eastern Cooperative Oncology Group performance status ≤ 2.

Patients were not eligible to participate in the study if they had previously received disease-modifying agents for MDS (e.g., lenalidomide, hypomethylating agents), luspatercept, or an allogeneic or autologous hematopoietic cell transplant. Other exclusion criteria included a diagnosis of myelodysplastic/myeloproliferative neoplasms according to the WHO 2016 classification (i.e., chronic myelomonocytic leukemia, atypical chronic myeloid leukemia, BCR-ABL1 negative, juvenile myelomonocytic leukemia) [23].

### Study treatment and procedures

Luspatercept was administered to patients as a subcutaneous injection once every 3 weeks for ≥ 24 weeks unless the patient met any of the treatment discontinuation criteria. The luspatercept starting dose was 1.0 mg/kg. To maintain the hemoglobin level within a target range of 10–12 g/dL (6.2–7.5 mmol/L) without RBC transfusions, starting from week 7 (i.e., dose 3), the luspatercept dose could be increased in a stepwise manner, first to 1.33 mg/kg, up to a maximum of 1.75 mg/kg.

In the event of a grade ≥ 3 treatment-related treatment-emergent adverse event (TEAE), luspatercept dosing was delayed until the TEAE resolved to grade ≤ 1, at which point a reduced dose was administered. A reduced dose was also required if, compared with the pre-dose level of the previous cycle, the hemoglobin level increased by > 2.0 g/dL. If a patient’s pre-dose hemoglobin level was ≥ 12 g/dL, a dose delay was required until the hemoglobin level was < 11.0 g/dL.

All patients underwent an MDS disease assessment on day 169 and every 24 weeks thereafter. Patients could continue to receive luspatercept after day 169 if they had evidence of clinical benefit, which was noted by either remaining NTD or having an International Working Group (IWG)-defined hematological improvement-erythroid (HI-E) response (≥ 1.5 g/dL increase in hemoglobin level for ≥ 8 weeks) within the 12 weeks before day 169 and every 24 weeks thereafter, and if they did not have disease progression according to IWG criteria [25]. Patients who did not have evidence of clinical benefit or had disease progression discontinued luspatercept and entered post-treatment follow-up. Patients could receive best supportive care, including RBC transfusions, antibiotics, antivirals, and antifungals, as needed.

In the first 10 patients to be enrolled in the study, blood samples for pharmacokinetic analyses were collected on days 1 and 3 of weeks 1 and 2; day 1 of weeks 3, 4, 10, 13, 16, 17, 18, 19, and 22; at the 24-week MDS disease assessment visit; and every 12 weeks thereafter for up to 1 year from the first dose (dense sample collection schedule). For the subsequent 10 patients, blood samples were collected on day 1 of weeks 1, 2, 3, 4, 10, 16, and 22; at the 24-week MDS disease assessment visit; and every 12 weeks thereafter for up to 1 year from the first dose (sparse sample collection schedule). Serum concentrations of luspatercept were analyzed using a validated enzyme-linked immunosorbent assay.

All patients who discontinued treatment with luspatercept entered long-term follow-up, during which information was collected on subsequent MDS therapies, progression to AML and other malignancies/pre-malignancies, and overall survival. Patients were followed for 5 years from the date of the first dose of luspatercept or for 3 years from the last dose, whichever occurred later, or until they died, were lost to follow-up, or withdrew consent.

### Endpoints

The primary endpoint of the study was the proportion of patients who achieved HI-E response per IWG criteria (≥ 1.5 g/dL increase in hemoglobin level for a consecutive 8-week period [25]) in the absence of RBC transfusions within the first 24 weeks of luspatercept treatment. In the event of dose delays because of a pre-dose hemoglobin level ≥ 12 g/dL, the time from dose delay to achieving an increase in hemoglobin of ≥ 1.5 g/dL after treatment resumption was included in the period of sustained hemoglobin increase even if there was a temporary decrease in hemoglobin to < 1.5 g/dL.

Secondary endpoints included the proportion of patients who achieved HI-E response within 48 weeks in the absence of RBC transfusions; the proportion of patients who achieved modified HI-E (mHI-E) response (≥ 1.5 g/dL mean increase in hemoglobin level for ≥ 8 weeks) in the absence of RBC transfusions within the first 24 and 48 weeks of treatment; time to and duration of HI-E and mHI-E response; the proportion of patients who remained NTD (no RBC transfusions during the treatment period) at weeks 24, 48, and 72; and the safety and pharmacokinetic profiles of luspatercept.

Safety was evaluated by the incidence of TEAEs until 42 days after the last dose and their causality to luspatercept (treatment-related TEAEs), as well as by the frequency of TEAEs leading to death, or to discontinuation or dose modification of luspatercept. Selected TEAEs of special interest included asthenia, hypertension, pre-malignant disorders, renal toxicity, immunogenicity type reactions, thromboembolic events, malignancies, extramedullary hematopoiesis masses, and liver toxicity. TEAEs of special interest were defined on the basis of non-clinical findings or the known safety profile [20, 22, 26–29] of the study drug. TEAEs were encoded using the Medical Dictionary for Regulatory Activities version 25.0 and presented as the worst grades based on the Common Terminology Criteria for Adverse Events version 4.03.

### Statistical analysis

For the primary efficacy analysis, the null hypothesis was that the proportion of patients who would achieve HI-E response by week 24 would be ≤ 10%. This proportion was based on the results of ARCADE, a phase 3 randomized placebo-controlled trial conducted in European countries, in which the HI-E response rate for patients with IPSS-defined low- or intermediate-1 risk MDS treated with darbepoetin-α was 14.7% (95% confidence interval [CI], 7.6–24.7) by week 24 [13]. In the present trial, 45% of patients were expected to achieve HI-E response based on the results of the phase 2 PACE-MDS dose-finding trial, in which the preliminary HI-E response rate was 63% (95% CI, 48–76) for patients with low- or intermediate-1 risk MDS treated with luspatercept 0.75–1.75 mg/kg [29]. With an expected HI-E response rate of 45%, a sample size of 20 patients would provide 95% power with a one-sided significance α of 0.025, based on a one-sample binomial exact test for the null hypothesis, which would be rejected if the lower limit of the 95% CI for the HI-E response rate was > 10%.

Exploratory analyses were performed for the primary endpoint across predefined patient subgroups: age (≤ 64 years, 65–74 years, ≥ 75 years); sex; RS status; baseline serum erythropoietin (≤ 200 U/L, > 200 U/L, ≤ 500 U/L, > 500 U/L); prior ESAs treatment; time since original MDS diagnosis (≤ 2 years, > 2 to ≤ 5 years, > 5 years); IPSS-R classification; splicing factor 3B subunit 1 (*SF3B1*) mutation status; and platelet count (< 100 × 10^9^/L, 100–450 × 10^9^/L, > 450 × 10^9^/L).

Secondary endpoints were summarized descriptively. Durations of HI-E and mHI-E response were estimated using the Kaplan–Meier method. Pharmacokinetic parameters within the first treatment cycle were determined using non-compartmental analyses of serum luspatercept concentrations from samples collected according to the dense schedule after the first dose. In addition, pharmacokinetic analyses were based on a one-compartment model with first-order absorption and elimination and were conducted using all available samples.

All efficacy analyses were performed on the intention-to-treat (ITT) population, defined as all enrolled patients. The pharmacokinetic population included all patients who received at least one dose of luspatercept and had sufficient samples collected and assayed for analysis. The safety population included all patients who received at least one dose of luspatercept.

Safety and efficacy analyses were conducted using SAS software, version 9.4 (SAS Institute, Cary, North Carolina, USA). Phoenix WinNonlin software, version 8.2 (Certara, Princeton, New Jersey, USA) was used for pharmacokinetic analyses.

## Results

### Patient disposition and baseline characteristics

Between May 2019 and November 2021, a total of 21 patients were enrolled and treated with luspatercept (Fig. 1). As of 1 July 2022 (data cut-off date for the primary analysis; median follow-up, 14.5 months), 17 (81.0%) and 10 (47.6%) patients had completed 24 and 48 weeks of treatment, respectively. There were 12 patients (57.1%) still receiving luspatercept; 9 patients (42.9%) had discontinued treatment. The most common reason for discontinuation was progressive disease per IWG criteria (n = 5; 23.8%).

The median duration of luspatercept treatment was 48.0 weeks (range, 3.0–147.4). Luspatercept dose was increased from 1.0 to 1.33 mg/kg in 10 patients (47.6%), and from 1.33 to 1.75 mg/kg in 6 patients (28.6%). Luspatercept dose reduction was required in 5 patients (23.8%) because of an increase in hemoglobin of ≥ 2.0 g/dL from the previous dose (n = 3) or a TEAE (n = 2), and dose delays were necessary in 11 patients (52.4%) because of a pre-dose hemoglobin level of ≥ 12.0 g/dL (n = 8) or a TEAE (n = 3).

As shown in Table 1, the median age of patients was 75 years and approximately 90% of patients were aged ≥ 65 years. There were 13 male patients (61.9%). Almost three-quarters of the patient population (71.4%) had IPSS-R low-risk MDS. RS were present in 13 patients (61.9%), and 10 patients (47.6%) carried *SF3B1* mutations. At baseline, the endogenous serum EPO level (median, 86.5 U/L) was ≤ 500 U/L in 17 patients (81.0%) and ≤ 200 U/L in 14 patients (66.7%). The median baseline hemoglobin level was 8.8 g/dL.

**Table 1:** Baseline patient characteristics

Characteristic	N = 21
Age, median (IQR), years	75.0 (70.0, 80.0)
Age, n (%), years	
≤ 64	2 (9.5)
65–74	8 (38.1)
≥ 75	11 (52.4)
Sex, n (%)	
Male	13 (61.9)
Female	8 (38.1)
Time since original MDS diagnosis, median (IQR), months	9.7 (2.4, 22.6)
Time since original MDS diagnosis, n (%), years	
≤ 1	11 (52.4)
> 1 to ≤ 2	5 (23.8)
> 2 to ≤ 5	3 (14.3)
> 5	2 (9.5)
MDS diagnosis (WHO 2017, central review), n (%)	
MDS-MLD	8 (38.1)
MDS-RS-MLD	13 (61.9)
RS-positive, n (%)	13 (61.9)
*SF3B1* mutation-positive, n (%)	10 (47.6)
IPSS-R risk category (central review), n (%)	
Very low	0
Low	15 (71.4)
Intermediate	5 (23.8)
High or very high	0
Missing	1 (4.8)
Serum EPO, median (IQR), U/L	86.5 (65.3, 298.3)
Serum EPO, n (%), U/L	
≤ 200	14 (66.7)
> 200	7 (33.3)
≤ 500	17 (81.0)
> 500	4 (19.0)
Hemoglobin, median (IQR), g/dL	8.8 (8.0, 9.2)
Hemoglobin, n (%), g/dL	
< 8	5 (23.8)
≥ 8	16 (76.2)
Platelet count, median (IQR), × 10^9^/L	206.0 (126.0, 258.0)
Platelet count, n (%), × 10^9^/L	
< 100	4 (19.0)
100–450	16 (76.2)
> 450	1 (4.8)

*EPO* erythropoietin, IPSS-R revised International Prognostic Scoring System (2012), *IQR* interquartile range, *MDS-MLD* myelodysplastic syndrome with multilineage dysplasia, *MDS-RS-MLD* myelodysplastic syndrome with ring sideroblasts with multilineage dysplasia, *RS* ring sideroblast, *SF3B1* splicing factor 3B subunit 1, *WHO* World Health Organization

### Efficacy

**Fig. 2:**
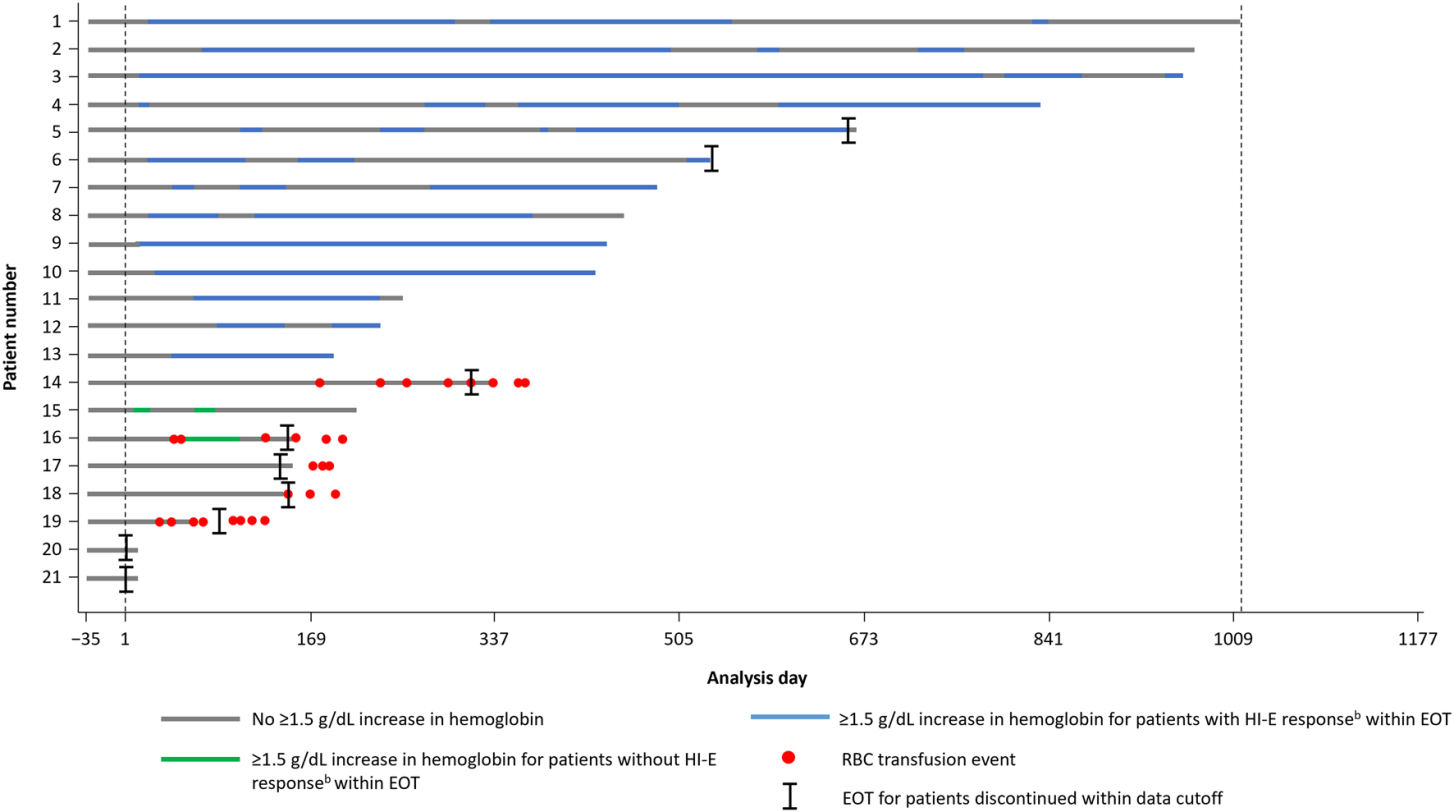
Duration of hematologic improvement (≥ 1.5 g/dL increase in hemoglobin vs baseline) and timing of RBC transfusions^a^ for patients treated with luspatercept (N = 21). ^a^Transfusion data collection will continue for ≥ 8 weeks after the last dose of luspatercept or until EOT (whichever is later). ^b^HI-E response: 1.5 g/dL increase in hemoglobin versus baseline sustained over any consecutive 56-day period in the absence of RBC transfusions. EOT, end of treatment; HI-E, hematologic improvement-erythroid; RBC, red blood cell

By week 24, HI-E response had occurred in 10 patients, resulting in a response rate of 47.6% (95% CI, 25.7–70.2; *P* < 0.0001; Table 2). The primary endpoint was met, and the null hypothesis was rejected. Among patients who achieved HI-E response by week 24, the median time to HI-E response was 26.5 days. Within 48 weeks, 12 patients (57.1%; 95% CI, 34.0–78.2) had achieved HI-E response. The Kaplan–Meier estimated median duration of response was 35.4 weeks (95% CI, 21.1–61.0; mean, 40.9 weeks). At weeks 24, 48, and 72, 17/21 patients (81.0%) in the ITT population remained NTD during luspatercept therapy (Fig. 2).

**Table 2:** Summary of efficacy outcomes

Endpoint	N = 21	
	HI-E	mHI-E
Response rate,^a^ % (95% CI)		
From day 1 of week 1 to week 24	47.6 (25.7, 70.2)*	57.1 (34.0, 78.2)
From day 1 of week 1 to week 48	57.1 (34.0, 78.2)	66.7 (43.0, 85.4)
Time to response,^b^ median (range), days	26.5 (14–85)	0.0^c^ (0–8)
Duration of response,^d^ median (95% CI), weeks	35.4 (21.1, 61.0)	65.1 (32.7, 137.4)

^a^Proportion of patients with a ≥ 1.5 g/dL increase (for HI-E) or ≥ 1.5 g/dL mean increase (for mHI-E) in hemoglobin versus baseline sustained over any consecutive 56-day period in the absence of red blood cell transfusions

^b^For patients who achieved HI-E or mHI-E response within 24 weeks

^c^Time to mHI-E response (time from day 1 of week 1 to first onset of mHI-E response) included day 1 of week 1 in all patients, resulting in a median value of 0

^d^Kaplan-Meier estimate for all patients who achieved HI-E or mHI-E response

^*^P < 0.0001

*CI* confidence interval, *HI-E* hematologic improvement-erythroid, *mHI-E* modified hematologic improvement-erythroid

**Fig. 3:**
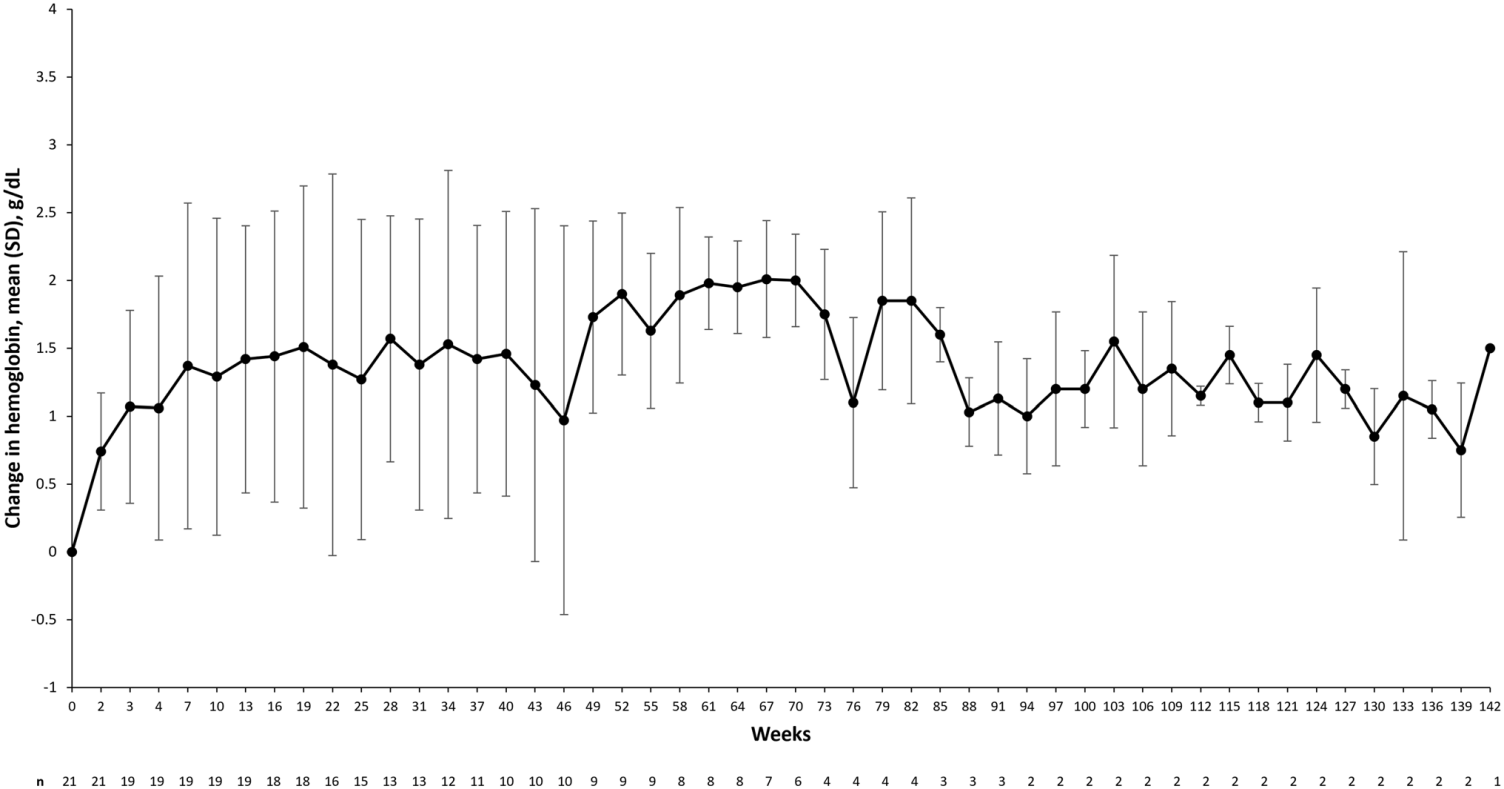
Mean change in hemoglobin from baseline at each study visit among patients receiving luspatercept treatment. SD, standard deviation

By week 24, mHI-E response had occurred in 12 patients (57.1%; 95% CI, 34.0–78.2; Table 2). At week 48, mHI-E response had occurred in an additional 2 patients, resulting in a response rate of 66.7% (95% CI, 43.0–85.4). The Kaplan–Meier estimated median duration of mHI-E response was 65.1 weeks (95% CI, 32.7–137.4; mean, 75.2 weeks). Mean change from baseline in hemoglobin level for patients receiving luspatercept is shown for each study visit in Fig. 3.

The efficacy of luspatercept was maintained across various patient subgroups (supplemental Fig. 2). Patients who carried the *SF3B1* mutation, were RS-positive, had baseline EPO level ≤ 200 U/L, or had been diagnosed within the previous 2 years tended to have the highest HI-E response rates among subgroups by week 24 (70.0% [95% CI, 34.8–93.3], 69.2% [38.6–90.9], 57.1% [28.9–82.3], and 56.3% [29.9–80.2], respectively) although the small sample size limits the interpretation of the results.

### Safety

Overall, TEAEs occurred in 20 patients (95.2%), and grade 3 or 4 TEAEs occurred in 7 patients (33.3%; supplemental Table 1). Treatment-related TEAEs occurred in 7 patients (33.3%), with grade 3 or 4 treatment-related TEAEs occurring in 3 patients (14.3%; Table 3). Grade 3 or 4 treatment-related TEAEs were hypertriglyceridemia, hyperuricemia, hypophosphatemia, and hypertension, each of which occurred in 1 patient (4.8%) and had improved to grade 1 or were resolved. There were no serious treatment-related TEAEs. No patients progressed to AML. There were no deaths during the study. TEAEs of special interest are shown in supplemental Table 2.

**Table 3:** Treatment-related treatment-emergent adverse events

Treatment-related TEAE, n (%)	N = 21	
	Any grade	Grade 3 or 4
Overall	7 (33.3)	3 (14.3)
Gastrointestinal disorders	2 (9.5)	0
Diarrhea	1 (4.8)	0
Stomatitis	1 (4.8)	0
Metabolism and nutrition disorders	2 (9.5)	2 (9.5)
Hypertriglyceridemia	1 (4.8)	1 (4.8)
Hyperuricemia	1 (4.8)	1 (4.8)
Hypophosphatemia	1 (4.8)	1 (4.8)
Eye disorders	1 (4.8)	0
Retinal hemorrhage	1 (4.8)	0
General disorders and administration site conditions	1 (4.8)	0
Injection site reaction	1 (4.8)	0
Infections and infestations	1 (4.8)	0
Cystitis	1 (4.8)	0
Investigations	1 (4.8)	0
Blood creatinine increased	1 (4.8)	0
Vascular disorders	1 (4.8)	1 (4.8)
Hypertension	1 (4.8)	1 (4.8)

*TEAE* treatment-emergent adverse event

Luspatercept was discontinued in 2 patients (9.5%) because of TEAEs considered to be unrelated to treatment (interstitial lung disease, panniculitis, and pneumonia [n = 1]; Alzheimer’s disease [n = 1]). In addition, two patients (9.5%) had treatment-related TEAEs that resulted in luspatercept dose reduction (hyperuricemia [n = 1]; hypertension [n = 1]). Five patients (23.8%) had TEAEs that resulted in the interruption of luspatercept treatment either for treatment-related TEAEs (hypophosphatemia and hypertriglyceridemia [n = 1]; hyperuricemia [n = 1]; blood creatinine increased [n = 1]; hypertension [n = 1]) or non-treatment-related TEAEs (lumbar vertebral fracture [n = 1]).

### Pharmacokinetics

Evaluable pharmacokinetic samples were obtained from 19 patients. Mean serum concentrations of luspatercept during the first treatment cycle are shown in supplemental Fig. 3.

Non-compartmental analysis revealed geometric mean values of 5.713 μg/mL (coefficient of variation [CV], 29.27%) for the maximum observed serum concentration (C_max_) of luspatercept, and 94.28 day × μg/mL (CV, 29.31%) for the area under the concentration–time curve (AUC) from day 1 to day 21. The median time to maximum observed serum concentration (T_max_) was 7.921 days (range, 2.88–13.75).

Compartmental analysis-derived pharmacokinetic parameters are shown in supplemental Table 3. According to this analysis, geometric mean AUC at steady state (AUC_tau,ss_), assessed over the 3-week dosing interval following the dose of 1.0 mg/kg every 3 weeks, and elimination half-life (t_1/2_) were 198.86 day × μg/mL (CV, 23.64%) and 14.08 days (CV, 94.70%), respectively.

## Discussion

Our phase 2, single-arm trial in 21 Japanese patients is the first to evaluate the efficacy, safety, and pharmacokinetics of luspatercept the treatment of anemia in NTD patients with LR-MDS. The prespecified primary analysis of the trial showed that luspatercept was associated with clinically and statistically significant improvements in anemia in these patients, and that it also had an acceptable safety profile.

In this trial, 47.6% of patients achieved the primary endpoint of IWG-defined HI-E response with the first 24 weeks of luspatercept treatment. This is a higher response rate than reported with darbepoetin-α in the phase 3 ARCADE trial in patients with IPSS-defined low- or intermediate-1 risk MDS (24-week HI-E response, 14.7%; 95% CI, 7.6–24.7) [13]. In this trial, the HI-E response rate was 57.1% among patients with a baseline serum EPO ≤ 200 U/L (n = 14), which is higher than that reported for NTD patients with LR-MDS and a baseline serum EPO level of < 200 U/L (20/40 patients [50.0%]) in the phase 3 EPOANE trial [14]. Additionally, 28.6% of patients with a baseline EPO > 200 U/L (n = 7) in this trial had a HI-E response, including patients who had an EPO level > 500 U/L, whereas in EPOANE, none of the patients with a baseline serum EPO level ≥ 200 U/L had an erythroid response (NTD and TD patients; n = 14) [14]. Collectively, these findings suggest that although baseline endogenous serum EPO concentration ≤ 200 U/L appears to be predictive of higher efficacy with ESA, luspatercept is a promising therapeutic option for the treatment of anemia in NTD patients with LR-MDS, regardless of serum EPO levels. In addition, HI-E response was observed across various subgroups, including RS subgroup, consistent with results from COMMANDS [21]. However, considering the small population size of the present trial, further research with larger patient numbers is required to confirm these findings.

In the PACE-MDS trial, the subgroup analysis of NTD patients (n = 34) showed the long-term efficacy of luspatercept, with an HI-E response rate of 70.6% after 5 years of follow-up [22]. In the present trial, the HI-E response rate increased from 47.6% at week 24 to 57.1% by treatment week 48. These data further support the long-term clinical benefit of luspatercept.

Most of the patients in this study were ESA-naïve (20/21 patients [95.2%]). Therefore, the HI-E responses observed in the current trial and the PACE-MDS trial [22, 29], together with a higher IWG-defined HI-E response rate observed with luspatercept compared with epoetin-α in ESA-naïve, TD patients in the COMMANDS trial (74% vs 51%; *P* < 0.0001) [21], suggests that treating LR-MDS patients with luspatercept earlier in the disease course prior to ESA therapy or RBC transfusions might be beneficial. Currently, the ongoing ELEMENT trial is assessing luspatercept versus epoetin-α in ESA-naïve NTD patients with LR-MDS [30].

In a systematic review that focused on the effectiveness and safety of ESAs [31], the authors noted the variations in erythroid response rate reported in different studies could be caused by the differences in definitions and evaluation criteria. In the present trial, HI-E response was defined by strictly applying the IWG 2006 criteria [25], in line with the previous phase 2 PACE-MDS trial of luspatercept and the phase 3 EPOANE trial, the registration trial that led to the approval of epoetin-α in MDS [14, 29]. In addition to the IWG definition of HI-E response, mHI-E was used as a secondary endpoint to ensure that the findings of the present trial could be easily compared with other studies [13, 20]. The use of mHI-E was expected to mitigate natural fluctuations in hemoglobin levels and any declines caused by treatment interruptions. Predictably, rates of mHI-E response were higher than rates of HI-E response (57.1% vs 47.6% at week 24 and 66.7% vs 57.1% at week 48, respectively) and the median duration of the mHI-E response was longer than that of the HI-E response (65.1 weeks vs 35.4 weeks, respectively). The higher rates and longer durations of mHI-E responses may suggest durable clinical benefits of luspatercept considering the fluctuation of hemoglobin levels in clinical practice.

In a previous publication of a population pharmacokinetic analysis in primarily white patients with MDS, the mean AUC_tau,ss_ and t_1/2_ of luspatercept 1.0 mg/kg were 151 day × μg/mL and 13 days, respectively [32]. In the current analysis of Japanese patients, the AUC_tau,ss_ and t_1/2_ of luspatercept (198.86 day × μg/mL and 14.08 days, respectively) were similar to those in non-Japanese patients, suggesting that there are no clinically meaningful differences in the pharmacokinetics of luspatercept in Japanese and non-Japanese patients with MDS. Furthermore, the safety outcomes in this trial were generally consistent with the known safety profile of luspatercept in patients with LR-MDS [20, 21, 29]. Of note, asthenia was only reported in one patient, giving a lower incidence than the previous studies [20, 21]. No new safety signals were identified.

The present trial had certain limitations, including its small sample size, which may limit the generalizability of the findings. Caution is particularly advised when interpreting the findings of subgroup analyses. Given the small numbers of patients in certain subgroups, including RS-negative patients, and patients with baseline serum EPO levels > 200 or > 500 U/L, meaningful conclusions are not possible. Furthermore, the trial was limited by its single-arm, open-label design.

## Conclusions

This trial was the first designed to demonstrate the clinical benefits of luspatercept in NTD patients with LR-MDS. The primary endpoint was met with 47.6% of patients achieving HI-E response within 24 weeks of starting therapy with luspatercept while remaining NTD. Although limited by a small sample size, the findings of this trial suggest that luspatercept is a promising therapy for NTD patients with LR-MDS. When begun early in the disease course, luspatercept may help to delay the need for RBC transfusions.

## Supplementary Information

Below is the link to the electronic supplementary material.

Supplementary file1 (DOCX 164 KB)

Supplementary file2 (PDF 391 KB)

Supplementary file3 (PDF 868 KB)

## Data Availability

Bristol Myers Squibb’s policy on data sharing may be found at https://www.bms.com/researchers-and-partners/clinical-trials-and-research/disclosure-commitment.html.
