# Supplementary material for: A phase 2 clinical trial of luspatercept in non-transfusion-dependent patients with myelodysplastic syndromes
Source: Int J Hematol. 2024 Nov 21;121(1):68–78. doi: 10.1007/s12185-024-03872-3 (PMC11741997; doi:10.1007/s12185-024-03872-3)

**A phase 2 clinical trial of luspatercept in non-transfusion-dependent patients with myelodysplastic syndromes**

**Authors:** Kosugi et al.

**Correspondence:** Hiroshi Kosugi, Department of Hematology, Ogaki Municipal Hospital, 4-86, Minaminokawacho, Ogaki, Gifu, 503-8502, Japan; phone: +81 (0)584-81-3341; fax: +81 (0)584-75-5715, email: hkosugi@med.nagoya-u.ac.jp

# Supplemental Data

## Supplemental Table 1.

Treatment-emergent adverse events

| **TEAE, n (%)** | **N=21** | |
| --- | --- | --- |
|  | **Any grade** | **Grade 3 or 4** |
| Overall | 20 (95.2) | 7 (33.3) |
| Constipation | 4 (19.0) | 0 |
| Arthralgia | 3 (14.3) | 1 (4.8) |
| Contusion | 3 (14.3) | 0 |
| Fall | 3 (14.3) | 0 |
| Myelodysplastic syndrome | 3 (14.3) | 0 |
| Back pain | 2 (9.5) | 0 |
| Blood creatinine increased | 2 (9.5) | 0 |
| Conjunctivitis | 2 (9.5) | 0 |
| Hypertension | 2 (9.5) | 2 (9.5) |
| Edema peripheral | 2 (9.5) | 0 |
| Pyrexia | 2 (9.5) | 0 |
| Stomatitis | 2 (9.5) | 0 |
| Vomiting | 2 (9.5) | 0 |
| Acne | 1 (4.8) | 0 |
| Alcohol poisoning | 1 (4.8) | 1 (4.8) |
| Aspartate aminotransferase increased | 1 (4.8) | 0 |
| Blood bilirubin increased | 1 (4.8) | 0 |
| Bronchitis | 1 (4.8) | 1 (4.8) |
| Chondrocalcinosis pyrophosphate | 1 (4.8) | 0 |
| Cough | 1 (4.8) | 0 |
| Cystitis | 1 (4.8) | 0 |
| Decreased appetite | 1 (4.8) | 0 |
| Dehydration | 1 (4.8) | 0 |
| Dementia Alzheimer’s type | 1 (4.8) | 0 |
| Diarrhea | 1 (4.8) | 0 |
| Dizziness | 1 (4.8) | 0 |
| Epistaxis | 1 (4.8) | 0 |
| Gamma-glutamyltransferase increased | 1 (4.8) | 0 |
| Gastric polyps | 1 (4.8) | 0 |
| Gastroenteritis | 1 (4.8) | 0 |
| Gastroesophageal reflux disease | 1 (4.8) | 0 |
| Glaucoma | 1 (4.8) | 0 |
| Glomerular filtration rate decreased | 1 (4.8) | 0 |
| Headache | 1 (4.8) | 0 |
| Hepatitis B DNA assay positive | 1 (4.8) | 0 |
| Herpes zoster | 1 (4.8) | 0 |
| Hyperlipidemia | 1 (4.8) | 0 |
| Hypertriglyceridemia | 1 (4.8) | 1 (4.8) |
| Hyperuricemia | 1 (4.8) | 1 (4.8) |
| Hypokalemia | 1 (4.8) | 1 (4.8) |
| Hypophosphatemia | 1 (4.8) | 1 (4.8) |
| Injection site pruritis | 1 (4.8) | 0 |
| Injection site reaction | 1 (4.8) | 0 |
| Interstitial lung disease | 1 (4.8) | 1 (4.8) |
| Iron overload | 1 (4.8) | 1 (4.8) |
| Leukocytosis | 1 (4.8) | 0 |
| Loss of consciousness | 1 (4.8) | 1 (4.8) |
| Lumbar vertebral fracture | 1 (4.8) | 1 (4.8) |
| Malaise | 1 (4.8) | 0 |
| Nausea | 1 (4.8) | 0 |
| Neutrophil count decreased | 1 (4.8) | 0 |
| Ocular hyperemia | 1 (4.8) | 0 |
| Panniculitis | 1 (4.8) | 1 (4.8) |
| Pericoronitis | 1 (4.8) | 0 |
| Peripheral swelling | 1 (4.8) | 0 |
| Pneumonia | 1 (4.8) | 0 |
| Proctalgia | 1 (4.8) | 0 |
| Productive cough | 1 (4.8) | 0 |
| Pruritus | 1 (4.8) | 0 |
| Restlessness | 1 (4.8) | 0 |
| Retinal hemorrhage | 1 (4.8) | 0 |
| Seborrheic dermatitis | 1 (4.8) | 0 |
| Skin ulcer | 1 (4.8) | 0 |
| Spinal fracture | 1 (4.8) | 0 |
| Subdural hygroma | 1 (4.8) | 0 |
| Synovial cyst | 1 (4.8) | 0 |
| Taste disorder | 1 (4.8) | 0 |
| Tinnitus | 1 (4.8) | 0 |
| Upper respiratory tract infection | 1 (4.8) | 0 |
| Urticaria | 1 (4.8) | 0 |
| White coat hypertension | 1 (4.8) | 0 |

TEAE, treatment-emergent adverse event.

## Supplemental Table 2.

Treatment-emergent adverse events of special interest

| **TEAE of special interest, n (%)** | **N = 21** |
| --- | --- |
| Asthenia | 1 (4.8) |
| Hypertension | 2 (9.5) |
| Premalignant disorders | 4 (19.0) |
| Myelodysplastic syndrome | 3 (14.3) |
| Gastric polyps | 1 (4.8) |
| Kidney toxicity | 3 (14.3) |
| Blood creatine increased | 2 (9.5) |
| Glomerular filtration rate decreased | 1 (4.8) |
| Immunogenicity injection local type reactions | 2 (9.5) |
| Injection site pruritus | 1 (4.8) |
| Injection site reaction | 1 (4.8) |
| Immunogenicity hypersensitivity type reactions | 0 |
| Thromboembolic events | 0 |
| Malignancies | 0 |
| Extramedullary hematopoiesis masses | 0 |
| Liver toxicity | 0 |

TEAE, treatment-emergent adverse event.

## Supplemental Table 3.

Compartmental pharmacokinetic parameters for luspatercept

|  | **n=19** |
| --- | --- |
| C_max_, geometric mean (CV%), µg/mL | 6.294 (29.56) |
| T_max_, median (range), days | 6.337 (1.44–10.59) |
| AUC_tau,ss_, geometric mean (CV%), day×µg/mL | 198.86 (23.64) |
| t_1/2_, geometric mean (CV%), days | 14.08 (94.70) |
| CL/F, geometric mean (CV%), L/day | 0.3094 (22.94) |
| V/F, geometric mean (CV%), L | 6.284 (90.62) |
| k_01_, geometric mean (CV%), day^−1^ | 0.3902 (111.57) |

AUC_tau,ss_, area under the concentration-time curve at steady state over the 3-week dosing interval following the dose of 1.0 mg/kg every 3 weeks; CL/F, apparent total clearance; C_max_, maximum observed serum concentration; CV, coefficient of variation; k_01_, absorption rate constant; t_1/2_, terminal elimination half-life; T_max_, time to maximum observed serum concentration; V/F, apparent volume of distribution.

## Supplemental Figure 1.

Study design


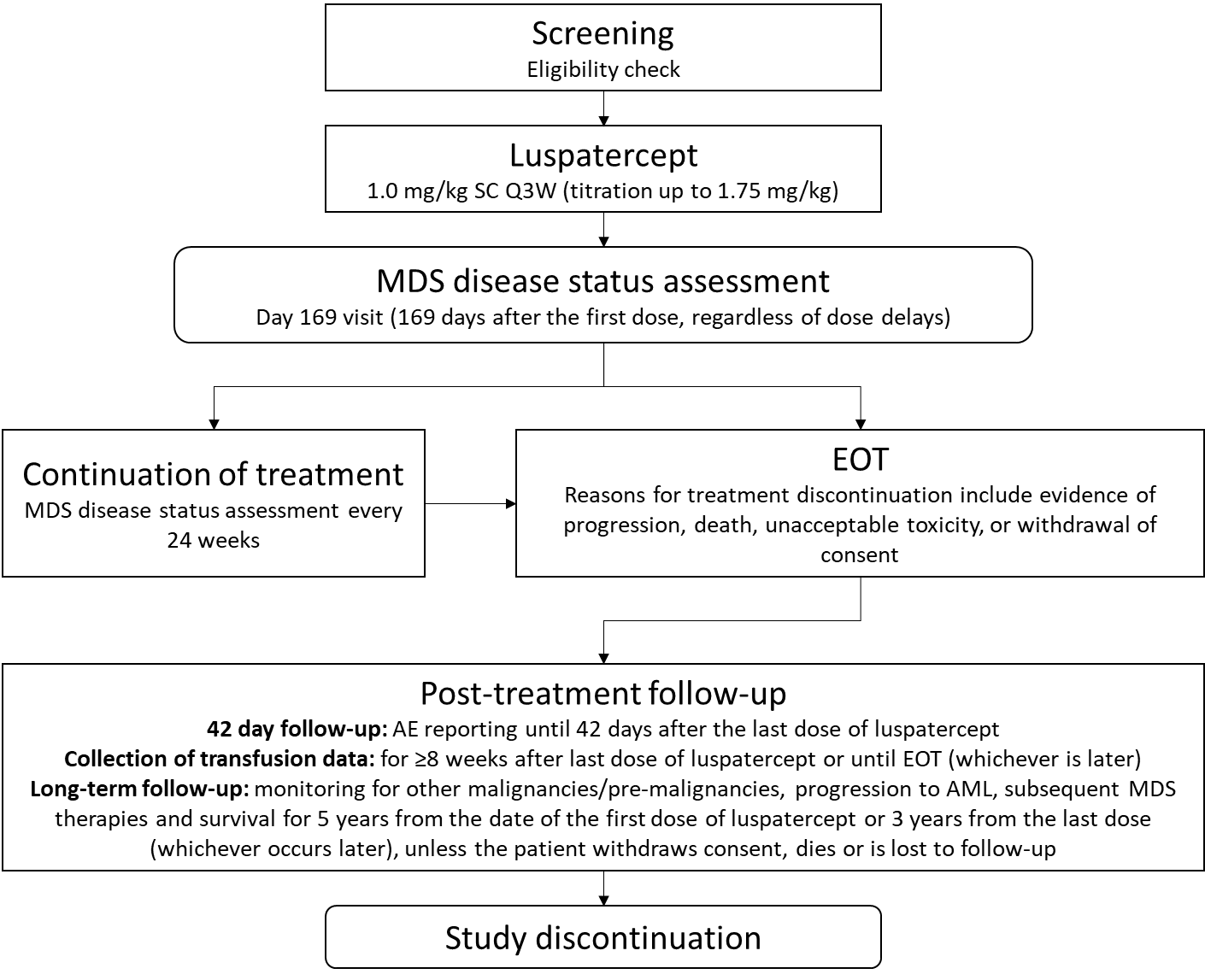


AE, adverse event; AML, acute myeloid leukemia; EOT, end of treatment; MDS, myelodysplastic syndrome; Q3W, once every 3 weeks; SC, subcutaneously.

## Supplemental Figure 2.

Forest plot of the subgroup analysis of HI-E response at week 24


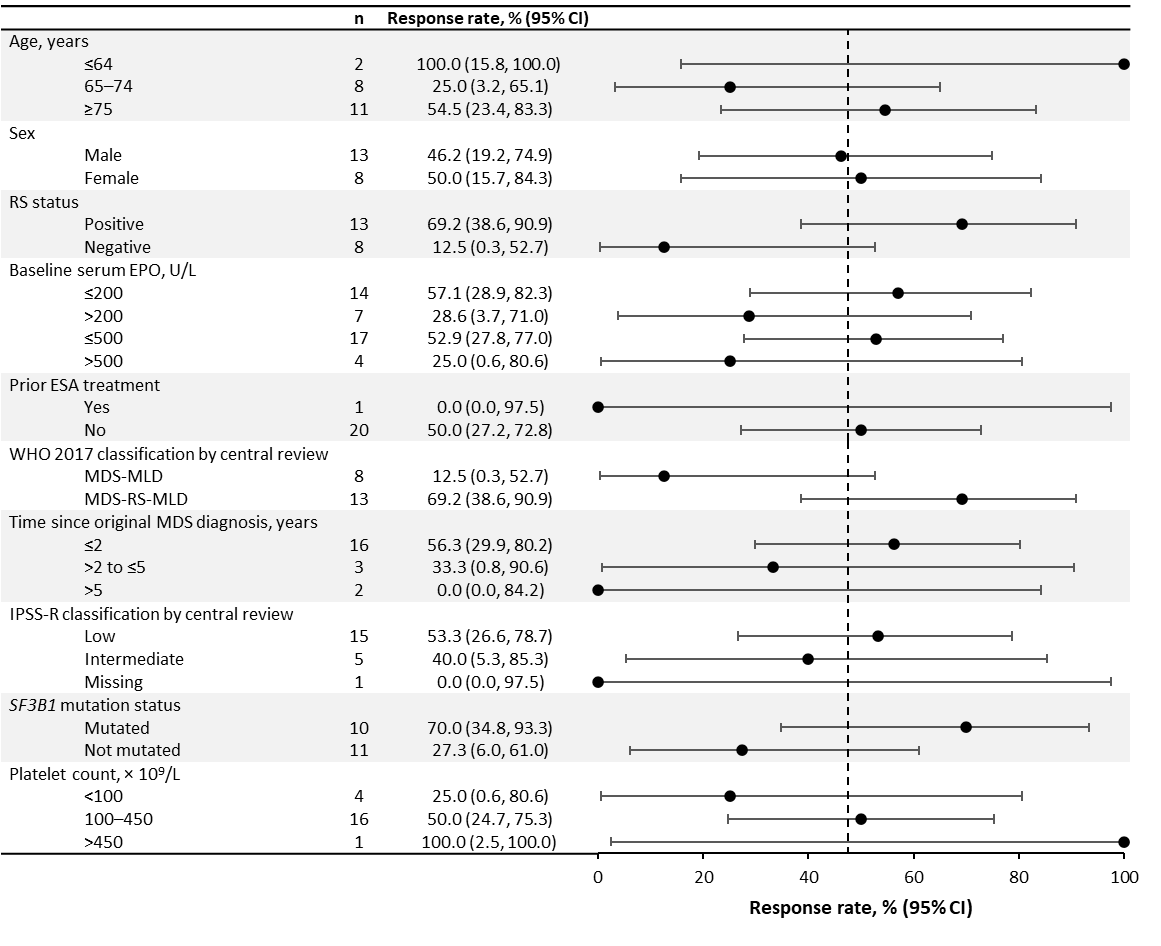


CI, confidence interval; ESA, erythropoiesis-stimulating agent; HI-E, hematologic improvement-erythroid; IPSS-R, revised International Prognostic Scoring System (2012); MDS, myelodysplastic syndrome; MDS-MLD, myelodysplastic syndrome with multilineage dysplasia; MDS-RS-MLD, myelodysplastic syndrome with ring sideroblasts with multilineage dysplasia; RS, ring sideroblast; *SF3B1*, splicing factor 3B subunit 1; WHO, World Health Organization.

## Supplemental Figure 3.

Mean serum luspatercept concentration during the first treatment cycle (n=19)


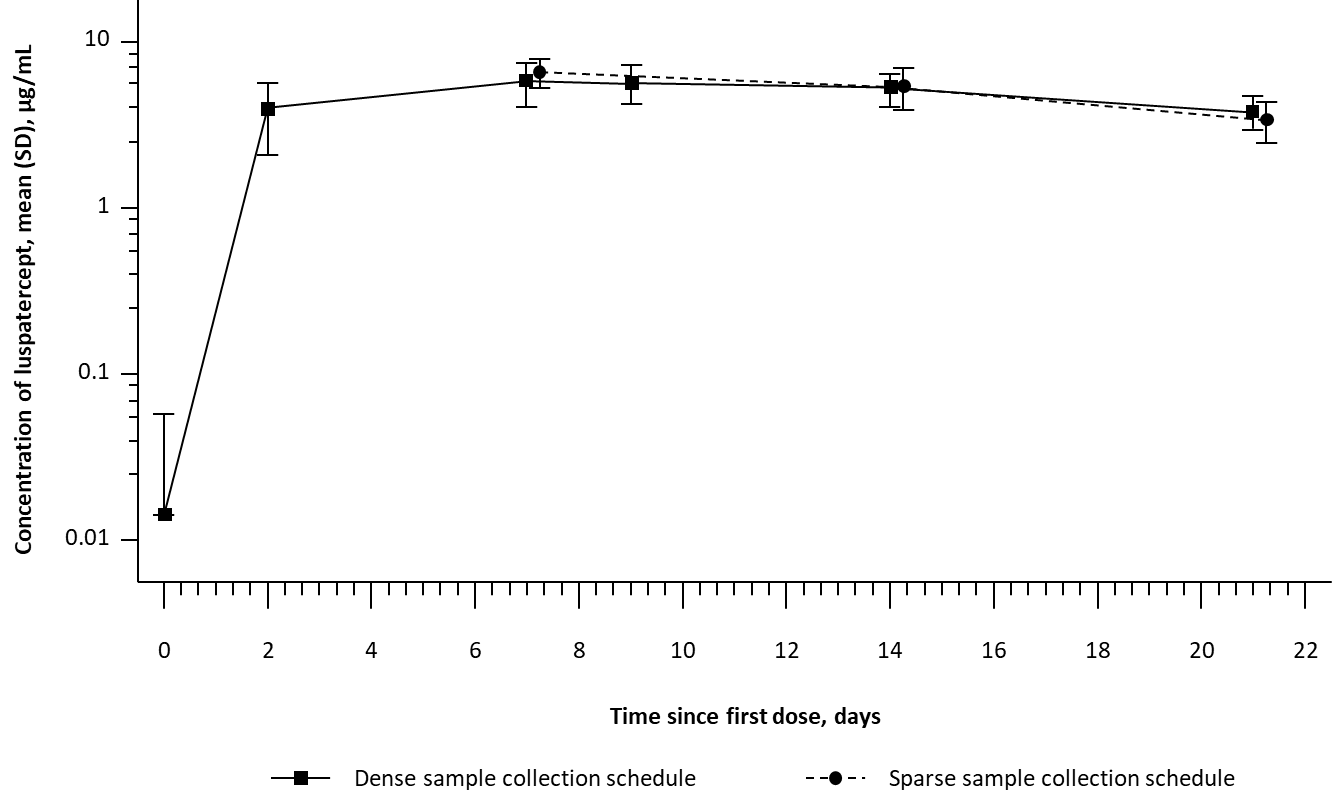

Supplement: Supplementary file 1 — Supplementary file1 (DOCX 164 KB) [file 12185_2024_3872_MOESM1_ESM.docx]
