# Supplementary material for: A phase 2 clinical trial of luspatercept in non-transfusion-dependent patients with myelodysplastic syndromes
Source: Int J Hematol. 2024 Nov 21;121(1):68–78. doi: 10.1007/s12185-024-03872-3 (PMC11741997; doi:10.1007/s12185-024-03872-3)
Supplement: Supplementary file 2 — Supplementary file2 (PDF 391 KB) [file 12185_2024_3872_MOESM2_ESM.pdf]

Publication Plain Language Summary:

A phase 2 clinical trial of luspatercept for the treatment of anemia in non-transfusion-dependent patients with myelodysplastic syndromes

The full title of the article is: A phase 2 clinical trial of luspatercept in non-transfusion-dependent patients with myelodysplastic syndromes.
Kosugi H, Fujisaki T, Iwasaki H, Shinagawa A, Iida H, Jo T, Kubonishi S, Morita Y, Nakashima Y, Onodera K, Suzuki K, Suzuki T, Tamai Y, Usuki K, Yokota A, Yonaga H, Hayakawa J, Midorikawa S, Nishio M, Suda M, Matsue K. *Int J Hematol* 2024.

This publication plain language summary (PPLS) has been developed to accompany the article and is not intended for any other use.
©Japanese Society of Hematology 2024

1 What was the purpose of the study?

The disease: what it is

Myelodysplastic syndromes (MDS) are a group of rare disorders in which blood cells in the bone marrow do not mature properly, causing the body to have fewer red blood cells than it needs (anemia). MDS can sometimes develop into acute myeloid leukemia (AML), which is an aggressive cancer of the white blood cells. There are different risk categories for MDS, and people with the “lower-risk” type are less likely to develop AML. The main symptom of lower-risk MDS is anemia, which can cause weakness, infections, and cardiovascular disease. Some people need blood transfusions to manage their anemia, and drugs can also help to reduce anemia and improve quality of life.

In people with lower-risk MDS who do not need blood transfusions and have low levels of erythropoietin (less than 500 U/L) (a hormone that stimulates red blood cell production), the currently recommended first treatment is a class of drugs known as erythropoiesis-stimulating agents (ESAs). Clinical trials have shown that ESAs do improve hemoglobin levels in these people, however, most will experience resistance and eventually stop responding to these drugs. At this point, there are few other treatment options available.

The treatment: how it works

Luspatercept is a drug known as an erythroid maturation agent (EMA), which helps red blood cells overcome the block that prevents them from maturing. A recent clinical trial has shown that in people with lower-risk MDS who were dependent on blood transfusions, luspatercept reduced anemia compared with an ESA. Results from another trial suggest that luspatercept might be especially effective in people with lower-risk MDS who were not dependent on blood transfusions.

The study: what it is about

This trial looked at how effective luspatercept is in treating anemia in people with lower-risk MDS who are not dependent on blood transfusions, and whether it causes any side effects. Japanese patients with MDS were enrolled in this study.

2 How was the study done?

This was a phase 2 trial, carried out at 16 clinical sites in Japan, that tested luspatercept in a small group of Japanese people with lower-risk MDS who were not dependent on blood transfusions and were eligible for the trial. The trial is ongoing (not yet complete).

The main result measured at the study (known as the primary endpoint) was the proportion of people who had an increase in hemoglobin level of 1.5 g/dL for 8 consecutive weeks (a measurement known as hematologic improvement-erythroid [HI-E] response), without needing a blood transfusion, within 24 weeks of starting luspatercept. When the proportion of people who achieved a HI-E response was more than 10%, the primary endpoint was considered to be met.

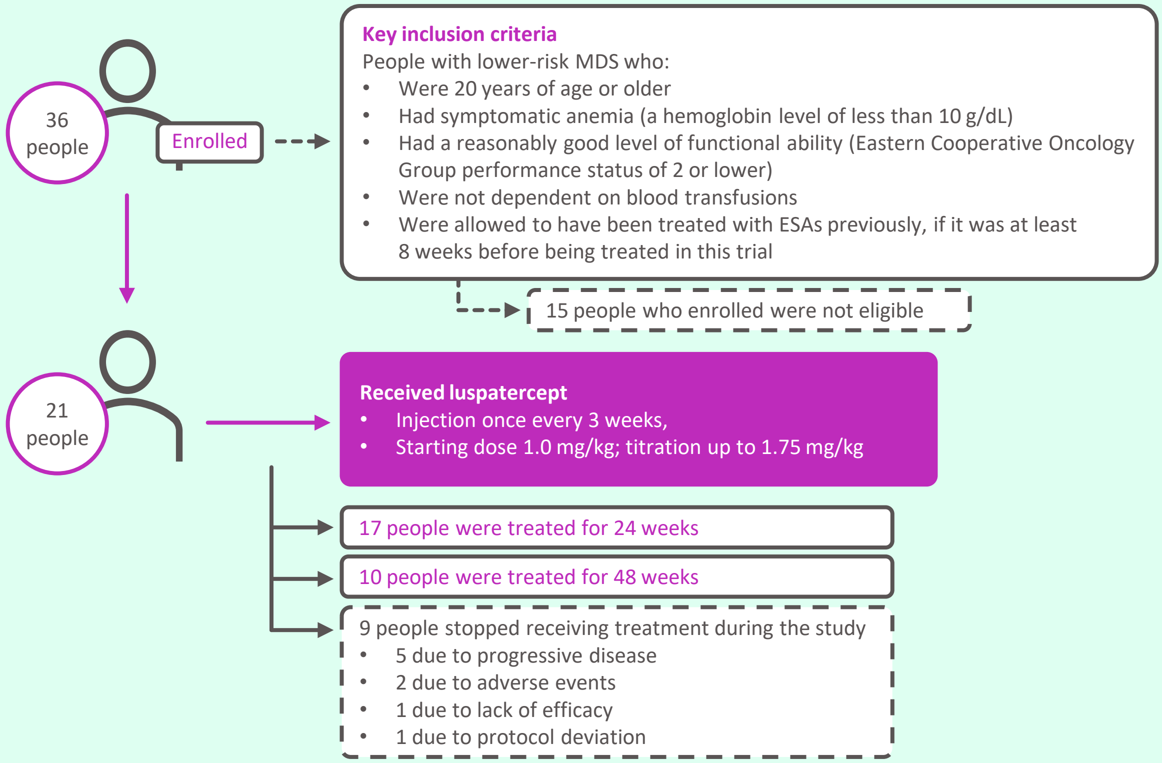

3 Who took part in the study?

Among the people who received luspatercept, almost two-thirds were male, nine-tenths were at least 65 years of age, and almost three-fourth had a baseline hemoglobin level higher than 8 g/dL.

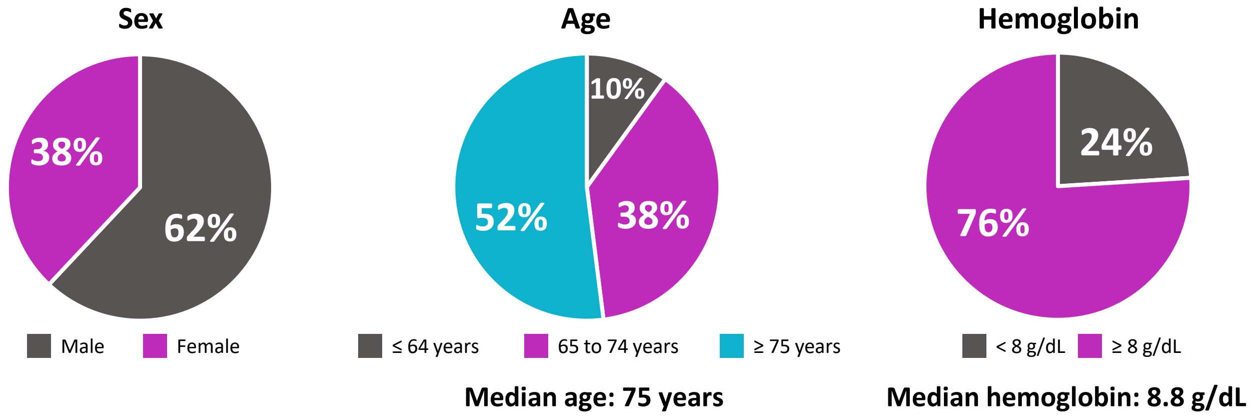

4 What were the main results of the study?

Effectiveness of luspatercept

After 24 weeks of treatment, the primary endpoint of the study was met, with 10 out of 21 people having a HI-E response (**48% of people responded to treatment**).

The median time it took for these people to achieve a HI-E response was **27 days**.

After 48 weeks of treatment, 12 people had achieved a HI-E response (**57% of people responded to treatment)**.

HI-E responses were shown to last a median of **35.4 weeks**.

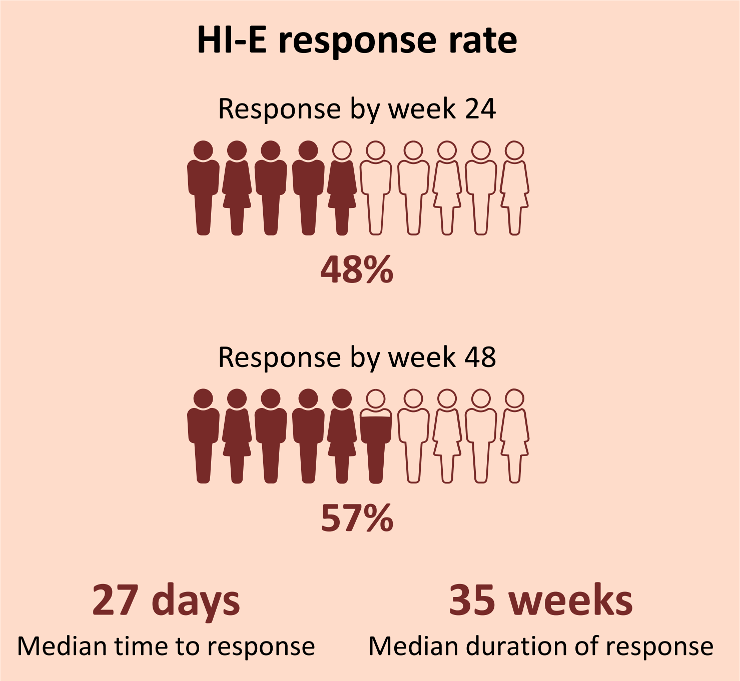

After starting the treatment with luspatercept, median hemoglobin levels in people changed as shown in the figure below.

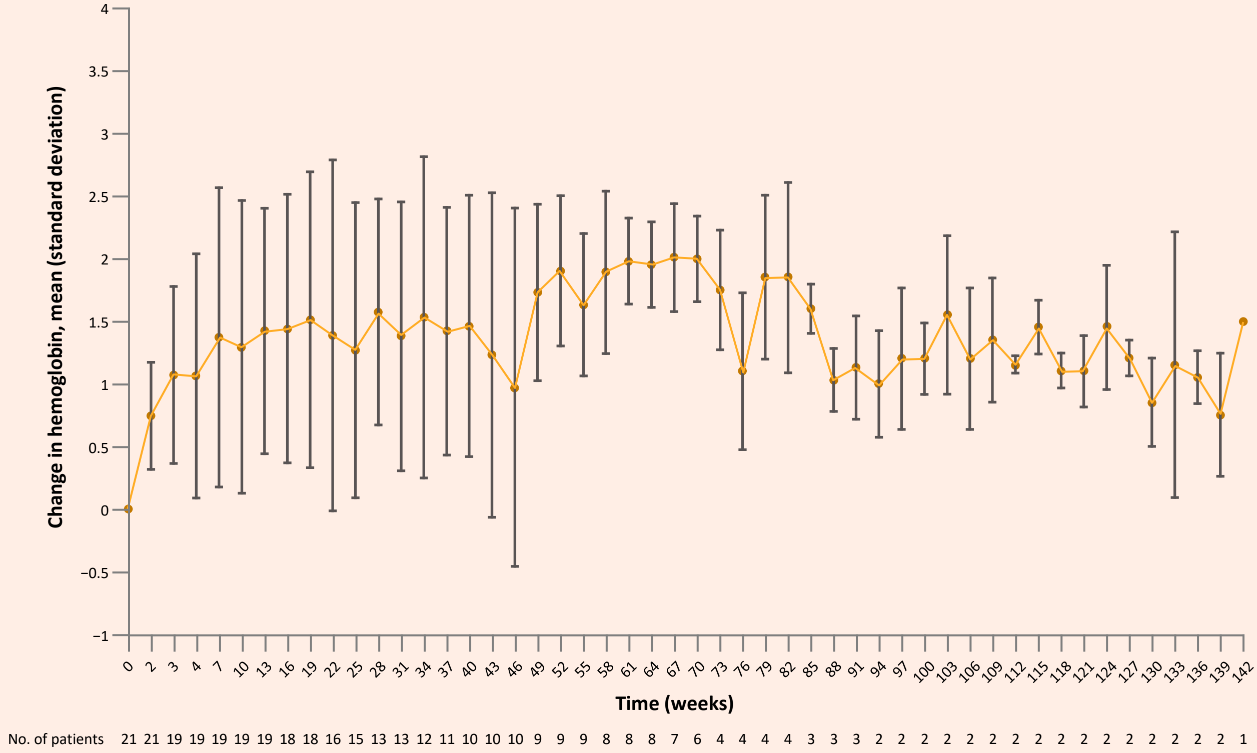

Safety of luspatercept

- Almost all (95%) of the people in this trial experienced treatment-emergent adverse events (TEAEs)[a]; one-third experienced severe TEAEs (grade 3 or 4).
- One-third of people in the trial experienced TEAEs thought to be related to luspatercept; in 3 of these people (14%), the TEAEs were classed as severe (grade 3 or 4).
- During the trial, no-one experienced serious TEAEs related to luspatercept (ie, that resulted in death, required inpatient hospitalization or the prolongation of hospitalization, were life-threatening, resulted in a persistent or significant disability/incapacity, or resulted in a congenital anomaly/birth defect), developed AML, or died.

TEAEs related to luspatercept

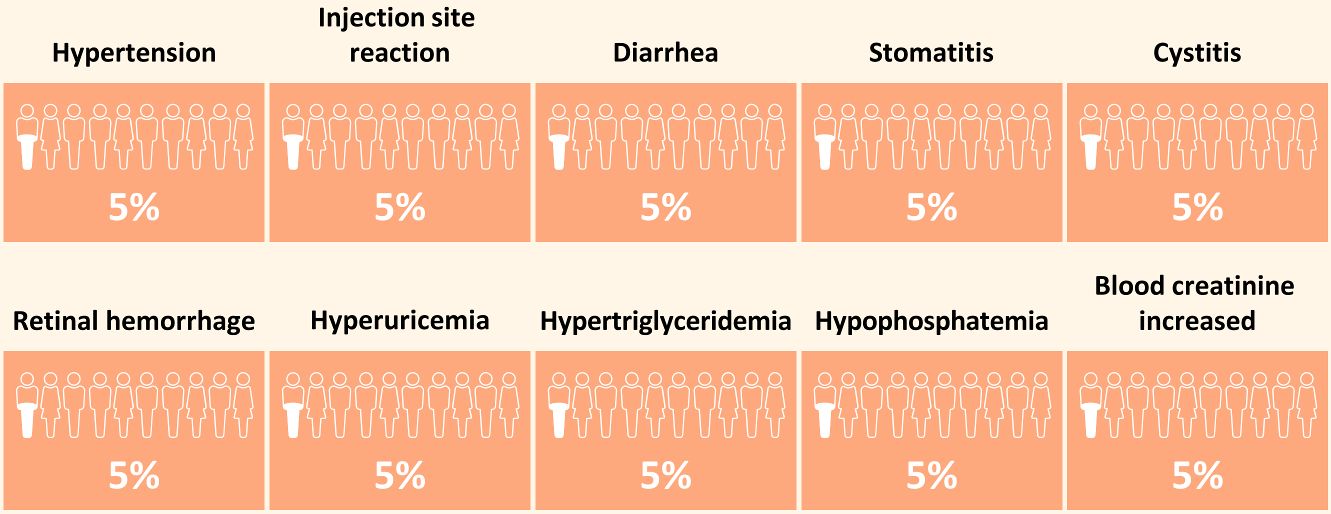

The severe TEAEs related to luspatercept (grade 3 or 4) were hypertension (5%), hyperuricemia (5%), hypertriglyceridemia (5%), and hypophosphatemia (5%).

[a]TEAEs were defined as any noxious, unintended, or untoward medical occurrence that may appear or worsen from the date the informed consent was signed until 42 days after the last dose of luspatercept

5 What were the main conclusions reported by the researchers?

This trial shows that luspatercept is a promising treatment for people with lower-risk MDS who are not dependent on blood transfusions.

- Almost half (48%) of the people treated with luspatercept achieved a HI-E response within 24 weeks of starting luspatercept treatment, without needing blood transfusions.
- The AEs related to luspatercept were generally consistent with those from previous clinical trials, and no new safety concerns were identified
- When people are treated with luspatercept early in the MDS disease course, it may delay the need for blood transfusions.
- Luspatercept treatment may help relieve anemia symptoms in these people

6 Who sponsored this study?

Medical writing assistance was funded by Bristol-Myers Squibb K.K. This study was supported by research funding from Bristol-Myers Squibb K.K.

Bristol Myers Squibb would like to thank everyone who took part in the study

7 Where can I find further information?

Further information about this study can be found in the links below:

https://clinicaltrials.gov/study/NCT03900715

Please see the original article for full author disclosure information

Plain language writing and graphical assistance was provided by Emma Rathbone, PhD at **Excerpta Medica**, and was funded by **Bristol-Myers Squibb K.K**.
