# Supplementary material for: A phase 2 clinical trial of luspatercept in non-transfusion-dependent patients with myelodysplastic syndromes
Source: Int J Hematol. 2024 Nov 21;121(1):68–78. doi: 10.1007/s12185-024-03872-3 (PMC11741997; doi:10.1007/s12185-024-03872-3)
Supplement: Supplementary file 3 — Supplementary file3 (PDF 868 KB) [file 12185_2024_3872_MOESM3_ESM.pdf]

出版物の平易な言葉による要約

骨髄異形成症候群に伴う貧血の治療薬ルスパテルセプトを輸血を必要としない患者さんで評価した第2相臨床試験

論文本編の書誌事項
A phase 2 clinical trial of luspatercept in non-transfusion-dependent patients with myelodysplastic syndromes.
Kosugi H, Fujisaki T, Iwasaki H, Shinagawa A, Iida H, Jo T, Kubonishi S, Morita Y, Nakashima Y, Onodera K, Suzuki K, Suzuki T, Tamai Y, Usuki K, Yokota A, Yonaga H, Hayakawa J, Midorikawa S, Nishio M, Suda M, Matsue K. *Int J Hematol* 2024.

この「出版物の平易な言葉による要約」は、上記の論文の付属資料として取り扱われることを意図して制作されており、それ以外の目的で使用されることを想定していません。©Japanese Society of Hematology 2024

1 この臨床試験を実施した目的は何ですか？

対象疾患：どのような病気ですか？

骨髄異形成症候群は、血液細胞が骨髄（骨の中心部にあり、赤血球を含む血液細胞が作られる場所）で十分に成熟しなくなることを特徴とする希少疾患の一つで、体に必要な赤血球が行き渡らない状態（貧血）となります。また、白血球ががん化して急性骨髄性白血病に移行することもあります。骨髄異形成症候群は予後（病気や治療などの医学的な経過についての見通し）のリスクの高さによって分類されており、“低リスク”の患者さんは急性骨髄性白血病に移行する可能性が低いことが知られています。低リスク骨髄異形成症候群でよくみられる症状は貧血で、貧血が引き金となって虚弱、感染症、心血管系の病気が起こることもあります。患者さんの中には貧血症状の改善のために輸血が必要となる方もいます。また、貧血症状の低減、生活の質（QOL）の改善のために薬物治療も行われています。

輸血を必要としない低リスク骨髄異形成症候群の患者さんで、かつエリスロポエチン（赤血球の産生を促進するホルモン）の値が500 U/Lを超えていない場合は、現在初回治療として赤血球造血刺激因子製剤に分類される薬剤を使用することが推奨されています。臨床試験では、赤血球造血刺激因子製剤による治療でこれらの患者さんのヘモグロビン値が実際に改善することが示されましたが、大部分の患者さんがその治療への抵抗性を獲得し、いずれその治療薬の効果は失われます。この状況に至ると、他に実施できる治療選択肢は限られています。

治療薬：どのように作用するのですか？

ルスパテルセプトは赤血球成熟促進薬に分類される薬剤の一つで、赤血球の成熟を抑えている一連の因子の働きを弱め、赤血球の成熟が進むように作用します。 近年実施された臨床試験では、輸血が必要な低リスク骨髄異形成症候群の患者さんにルスパテルセプトを投与したところ、赤血球造血刺激因子製剤を投与された場合と比較して貧血症状がより改善したことが示されています。別の臨床試験では、ルスパテルセプトは輸血の必要がない低リスク骨髄異形成症候群の患者さんに対して特に有効である可能性が示唆されています。

臨床試験：どのような試験を実施したのですか?

この試験では、ルスパテルセプトが輸血を必要としない低リスク骨髄異形成症候群の患者さんの貧血の治療にどの程度有効なのか、どのような副作用が生じるのかを調査しました。試験には日本人の骨髄異形成症候群の患者さんが参加しました。

② 臨床試験はどのように行われたのですか？

この試験は日本にある16の医療施設が参加した第2相試験です。輸血の必要がない低リスク骨髄異形成症候群の日本人患者さんで構成された少人数のグループに、ルスパテルセプトを投与して有効性と副作用を評価しました。この試験はまだ終了しておらず、現在も継続して評価が行われています。

試験で評価する最も重要な項目は主要評価項目と呼ばれます。本試験の主要評価項目は、輸血を必要とせず、かつルスパテルセプト投与開始から24週目までにヘモグロビン値が8週間連続して1.5 g/dLよりも上昇（赤血球系改善）した患者さんの割合、と設定されました。この赤血球系改善を示した患者さんの割合が統計学的に10%を超えた場合に、主要評価項目を達成したと定義されました。

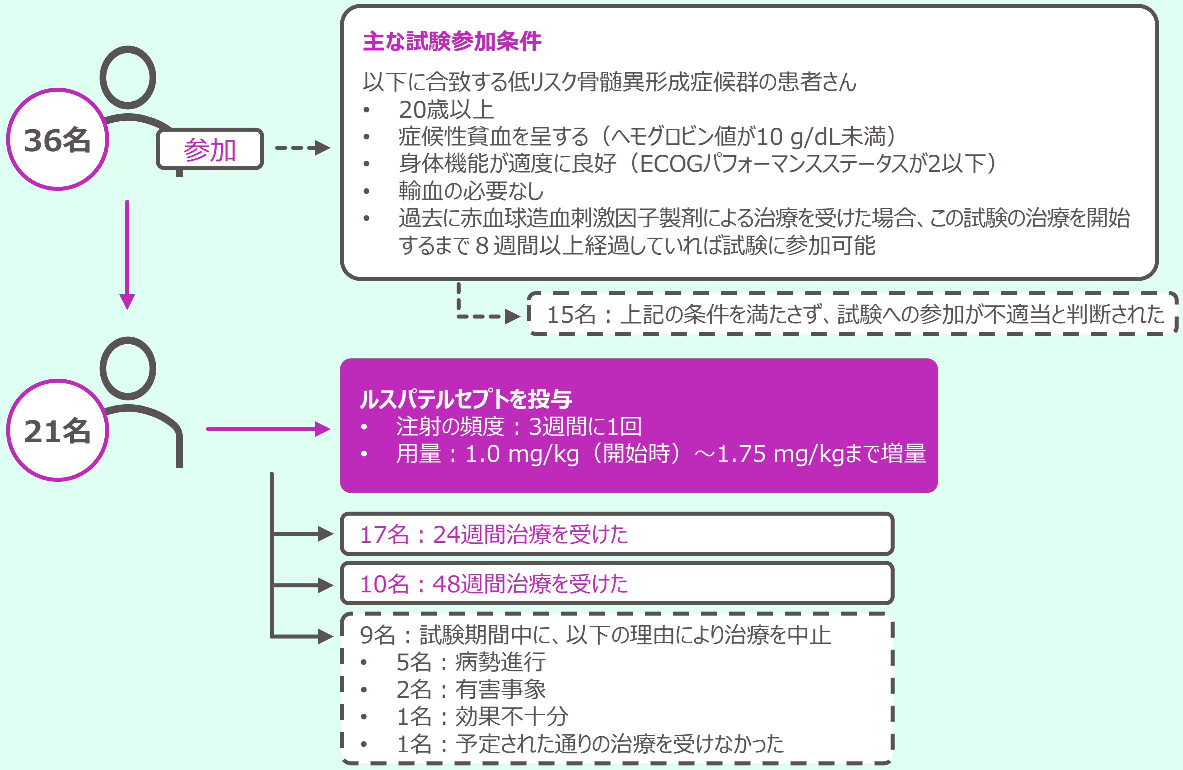

③ どのような患者さんが臨床試験に参加したのですか？

ルスパテルセプトを投与された患者さんのうち、約3分の2は男性、約10分の9は65歳以上、約4分の3は治療開始前のヘモグロビン値が8 g/dL以上でした。

性別

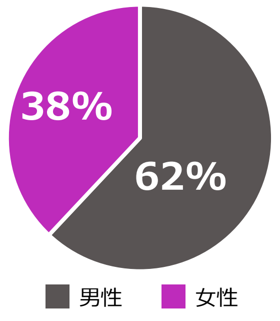

年齢

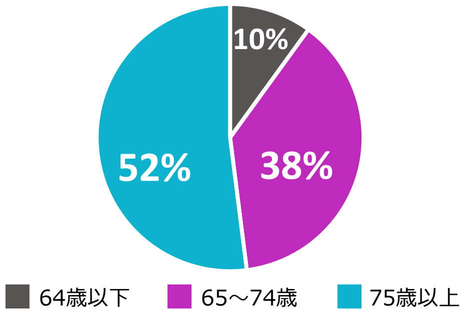

ヘモグロビン

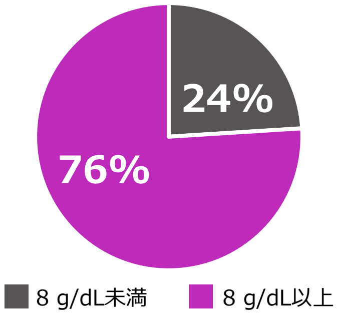

年齢（中央値）：75歳

ヘモグロビン（中央値）：8.8 g/dL

④ この臨床試験で得られた重要な結果は何ですか？

ルスパテルセプトの有効性

21名の患者さんのうち10名（**48%**）で、治療開始から24週間後までに赤血球系改善が認められ、主要評価項目を達成しました。

これらの患者さんが赤血球系改善を示すまでの期間の中央値は**27日**でした。

また、治療開始から48週間後には、12名（**57%**）で赤血球系改善が認められました。

赤血球系改善が続いた期間の中央値は**35.4週間**でした。

赤血球系改善率

24週までの結果

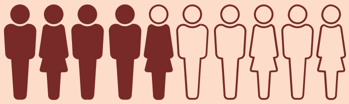

48%

48週までの結果

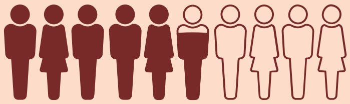

57%

27日
赤血球系改善までの期間（中央値）

35週間
赤血球系改善が続いた期間（中央値）

ルスパテルセプトの投与開始後、患者さんのヘモグロビン値の平均値は以下のように変化しました。

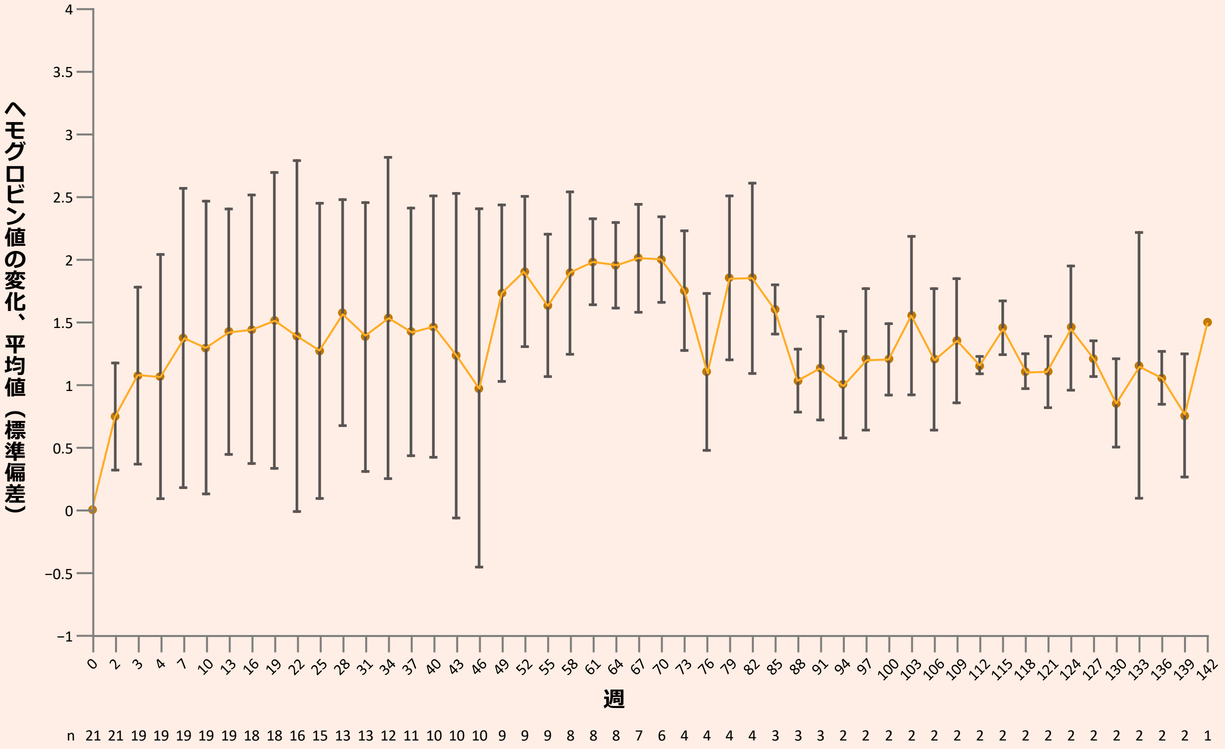

ルスパテルセプトの安全性

- 有害事象[a]はルスパテルセプトを投与された患者さんのほぼ全員（95%）で発生しました。また、重度の有害事象（グレード 3/4）は患者さん全体の3分の1で認められました。
- 副作用（有害事象の中で、ルスパテルセプトの投与との関連性が疑われるもの）は、患者さん全体の3分の1で認められました。これらの患者さんのうち3名（患者さん全体の14%）の副作用は重度（グレード 3/4）であると判定されました。
- 試験期間中、重篤な副作用（死亡に至るもの、入院または入院期間の延長が必要なもの、生命を脅かすもの、永続的または顕著な障害・機能不全を引き起こすもの、先天異常を来すもの）は発生せず、急性骨髄性白血病への移行、死亡はいずれも発生しませんでした。

副作用

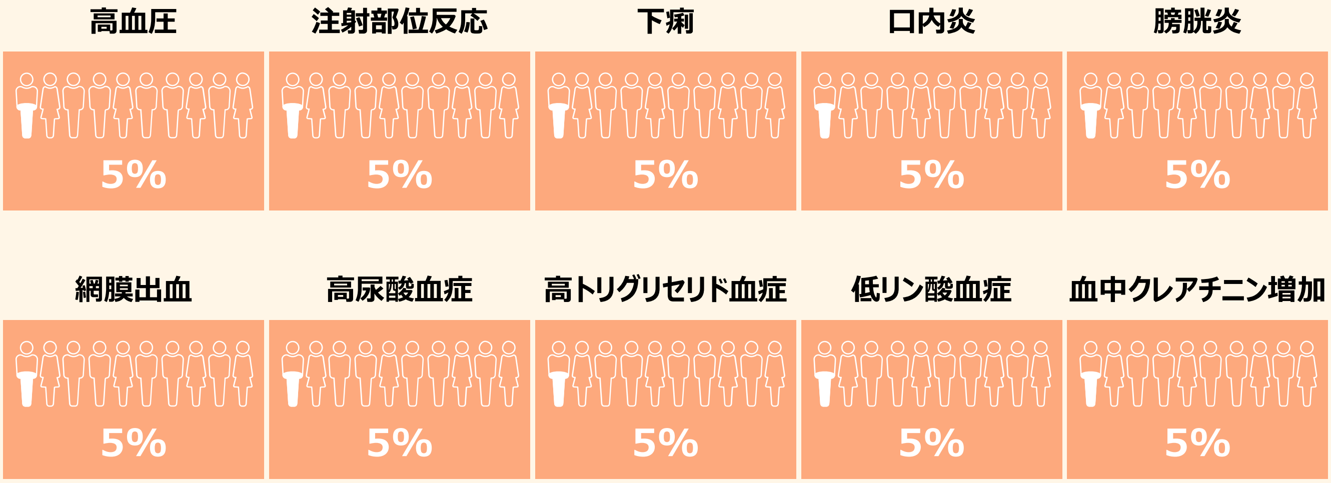

重度の副作用（グレード 3/4）としては、高血圧（5%）、高尿酸血症（5%）、高トリグリセリド血症（5%）、低リン酸血症（5%）が発生しました。

[a]本試験における有害事象は、同意取得からルスパテルセプト投与終了後42日までに患者さんに生じた又は悪化したあらゆる有害で意図しない、あるいは好ましくない医療上の出来事を指します。

5 この臨床試験の重要な結論は何ですか？

この試験によって、ルスパテルセプトは輸血を必要としない低リスク骨髄異形成症候群の患者さんに対して有望な治療薬であることが示されました。

- ルスパテルセプトを投与された患者さんの約半数（48%）は、投与開始から24週間後まで輸血を必要とすることなく、赤血球系改善が認められました。
- この試験で報告された副作用は、低リスク骨髄異形成症候群の患者さんを対象としたこれまでの試験と同様であり、新たな懸念は認められませんでした。
- ルスパテルセプトを骨髄異形成症候群の進行過程の初期段階で投与すると、輸血が必要な状態になるまでの期間を遅らせることができる可能性が示されました。
- ルスパテルセプトは骨髄異形成症候群の患者さんの貧血の症状の緩和に役立つ可能性が示されました。

6 この臨床試験は誰の依頼で実施されたのですか？

本稿の執筆にあたっては、ブリストル・マイヤーズ スクイブ株式会社の資金提供によりメディカルライティングサポートを受けました。また、本試験はブリストル・マイヤーズ スクイブ株式会社から研究費の提供を受けて実施されました。

ブリストル・マイヤーズ スクイブ株式会社は、この試験にご参加下さったすべての方々に御礼を申し上げます。

7 この臨床試験について詳しく知りたい場合、どこで情報を得られますか？

この臨床試験について詳しく知りたい場合は、以下のwebサイトで情報が公開されています。

https://clinicaltrials.gov/study/NCT03900715

著者の開示事項については、この要約のもととなった論文に記載されています。

この要約原稿の執筆および図版作成の補助は、**ブリストル・マイヤーズ スクイブ株式会社**の資金提供を受けて、Emma Rathbone（PhD、**Excerpta Medica**）が担当しました。
